# (E)-2-Methoxy-4-(3-(4-Methoxyphenyl) Prop-1-en-1-yl) Phenol Suppresses Breast Cancer Progression by Dual-Regulating VEGFR2 and PPARγ

**DOI:** 10.4014/jmb.2309.09019

**Published:** 2023-11-03

**Authors:** Na-Yeon Kim, Hyo-Min Park, Hee Pom Lee, Jin Tae Hong, Do-Young Yoon

**Affiliations:** 1 Department of Bioscience and Biotechnology, Konkuk University, Seoul 05029, Republic of Korea; 2 College of Pharmacy & Medical Research Center, Chungbuk National University, Cheongju 28644, Republic of Korea

**Keywords:** Breast cancer treatment, MMPP, PPARγ, VEGFR2, AKT, anti-cancer effect

## Abstract

In cancer treatment, multi-target approach has paid attention to a reasonable strategy for the potential agents. We investigated whether (E)-2-methoxy-4-(3-(4-methoxyphenyl) prop-1-en-1-yl) phenol (MMPP) could exert an anticancer effect by dual-regulating VEGFR2 and PPARγ. MMPP showed modulating effects in TNBC type (MDA-MB-231 and MDA-MB-468) and luminal A type (MCF7) breast cancer cell lines. MMPP enhanced PPARγ transcriptional activity and inhibited VEGFR2 phosphorylation. MMPP-induced signaling by VEGFR2 and PPARγ ultimately triggered the downregulation of AKT activity. MMPP exhibited anticancer effects, as evidenced by growth inhibition, inducement of apoptosis, and suppression of migration and invasion. At the molecular level, MMPP activated pro-apoptotic proteins (caspase3, caspase8, caspase9, and bax), while inhibiting the anti-apoptotic proteins (bcl2). Additionally, MMPP inhibited the mRNA expressions of EMT-promoting transcription factors. Therefore, our findings showed molecular mechanisms of MMPP by regulating VEGFR2 and PPARγ, and suggested that MMPP has potential to treat breast cancer.

## Introduction

Breast cancer (BC) is a prevalent and life-threatening diseases among women [1]. The development of anticancer drugs has been focused on specific targets with high potency for breast cancer [2]. However, single targeting may be insufficient when the targeted molecule is inactivated or inhibited, leading to drug resistance [3]. Therefore, dual/multi-target therapeutics are preferred for cancer therapy. In the breast cancer therapy, following strategies are commonly used: (1) apoptosis stimulation and (2) prevention of migration and invasion [4]. We focused on the development of a dual-target drug that can simultaneously satisfy these strategies.

Peroxisome proliferator-activated receptor gamma (PPARγ) regulates adipogenesis, and its activity is regulated by agonists [5]. However, PPARγ is also significantly expressed in breast cancer [6]. PPARγ agonists have been reported to have anticancer effects by inducing the transcription of phosphatase and tensin homolog deleted on chromosome 10 (PTEN), a well-known tumor suppressor [7]. Upregulated of PTEN inhibits the AKT pathway, leading to suppression of breast cancer survival, and induction of apoptosis. Several thiazolidinedione derivatives, which are PPARγ agonists, have been documented that it has potent anticancer effects by inhibiting vascular endothelial growth factor receptor 2 (VEGFR2) [8]. VEGFR2 is expressed in breast cancer and plays crucial part in promoting cell survival and migration [9]. Therefore, VEGFR2 is an effective target for breast cancer. In a previous study, we reported that (E)-2-methoxy-4-(3-(4-methoxyphenyl) prop-1-en-1-yl) phenol (MMPP) inhibits STAT3 and shows anti-cancer effects in non-small cell lung cancer [10]. Furthermore, MMPP is a PPARγ agonist in adipocytes and a VEGFR2 inhibitor in endothelial cells [11, 12]. Thus, MMPP possesses anti-inflammatory, anticancer, and anti-diabetic properties [10, 13]. However, its anticancer effects have yet to be determined in breast cancer. It is hypothesized that MMPP may exercise its effects on breast cancer by acting on VEGFR2 and PPARγ. The dual targeting on VEGFR2 and PPARγ is an effective strategy for breast cancer therapy [14]. As a result, the purpose of our study is to verify the regulatory effects of MMPP on VEGFR2 and PPARγ. As a dual-targeting drug, MMPP is expected to be an effective anticancer drug for preventing breast cancer while reducing drug resistance.

## Materials and Methods

### Reagents Preparation

MMPP was synthesized and provided by Dr. Jin Tae Hong (Chungbuk National University, Republic of Korea)[15]. Briefly, MMPP was synthesized via the Heck reaction using phenyl halide, allylbenzene, triphenylphosphine, Pd(OAc)_2_, and tributylamine. The mixture was purified using flash chromatography on silica gel in hexane and ethyl acetate (3:1, v/v).

### Cell Culture

All human cell lines were purchased from the American Type Culture Collection (ATCC, USA). MCF-7 (luminal A) and MDA-MB-231 (triple-negative breast cancer (TNBC); claudin-low) were cultured in DMEM, whereas MDA-MB-468 (TNBC; basal) were cultured in RPMI 1640 supplemented with 10% (v/v) thermally inactivated fetal bovine serum (Hyclone Laboratories, USA). To generate a 3D model, cells were seeded in ultra-low attachment round-bottom 96-well plates (Corning, USA) and allowed to form multicellular aggregates after 48 h [16, 17]. All cells were incubated at 37°C and 5% CO_2_.

### Examination of p-VEGFR2 Levels

In vitro, phosphorylation of VEGFR2 was assessed by a cell-based enzyme-linked immunoassay (ELISA) method [18]. The cells were seeded in 96-well plates and treated with MMPP for 30 min at 37°C. After fixing with 10% formalin, cells were incubated with anti-pVEGFR (Tyr 1175) antibody (1:1000) for 1 h. The cells were washed twice with PBST and incubated with anti-rabbit mouse IgG conjugated with HRP (1:5000) for 1 h. After washing, the cells were treated with TMB substrate (Thermo Fisher Scientific, USA). The reaction was stopped by adding 2N H_2_SO_4_ and the absorbance was measured at 450 nm.

### Transcriptional Activity Assay

PPARγ transcriptional activity was assessed using a plasmid expressing (PPAR response element (PPRE) 3)-thymidine kinase-luciferase reporter construct, as described previously [11]. Breast cancer cells were seeded in 24-well plates and transfected with (PPRE3)-tk-luciferase and *Renilla* luciferase control reporter vectors using jetOPTIMUS transfection reagent (Polyplus, France). After treatment with MMPP, the cells were harvested and conducted a Dual-Luciferase Reporter Gene Assay Kit (Promega, USA).

### Gene Expression Experiments

Total RNA was isolated using the easy-BLUE Total RNA Extraction Kit (iNtRON, Korea). First-strand cDNA was synthesized with M-MulV reverse transcriptase (New England Biolabs, USA). The synthesized cDNA was used for real-time polymerase chain reaction (RT-PCR) amplification of specific genes. The primers sequences are listed in Table S1.

### Immunoblot Analysis

Harvested cells were lysed in RIPA buffer to obtain whole protein extract. For nuclear and cytoplasmic fractionation, the cells were fractionated using NE-PER Nuclear and Cytoplasmic Extraction Reagents (Thermo Fisher Scientific Inc.), according to the manufacturer’s instructions. Equal amounts of quantified proteins were loaded on an appropriate percentage of SDS-PAGE gel and transferred onto a polyvinylidene fluoride (PVDF) blotting membrane (Cytiva, USA). The membranes were blocked with 5% skim milk for 1 h. Specific primary antibodies (1:1000) were incubated for 1 h. The primary antibodies were used against p-AKT1/2/3 (#sc-7985)(Santa Cruz Biotechnology, USA), PARP (#9542s), Caspase3 (#9662s), Caspase8 (#9746s), Caspase9 (#9502s), Bax (#2772s), Bcl2 (#15071s), and GAPDH (#sc-47724) (Cell Signaling Technology, USA). The membranes were probed with HPR-conjugated secondary anti-rabbit or anti-mouse IgG antibodies. Visualization was performed using ECL reagents (Advansta, USA).

### Cell Viability Assay

Cell viability was assessed using the 3-(4,5-dimethylthiazol-2-yl)-5-(3-carboxymethoxy phenyl)-2-(4-sulfophenyl)-2H-tetrazolium (MTS) and CellTiter 96 Aqueous One Solution Assays (Promega, USA). Briefly, 20 μl MTS reagent was added to each well and incubated for 1 h. The absorbance was measured at 492 nm.

### Colony Formation Assay

After seeding the cells in 6-well plates, the cells were treated with MMPP for 48 h. The medium was then changed into a fresh growth medium every other day for 5 days. Finally, the colonies were stained with Giemsa solution, and the colony areas were measured using ImageJ version 1.53t [19].

### Detection of Apoptosis

Annexin V-PI staining was assayed using Annexin V-FITC Apoptosis Detection Kit (BD Biosciences, USA). Briefly, the harvested cells were washed twice with phosphate buffered saline (PBS) and suspended in 1× binding buffer. The cells were labeled with Annexin V and PI for 15 min at a room temperature. At least 10,000 cells were analyzed using BD FACS Calibur Flow Cytometry System (BD Biosciences) to determine the percentage of apoptotic cells.

### Wound Healing Assay

Cells were seeded into 24-well plates, and allowed to grow until they are confluent. A 200 μl tip was used to scratch for wound area. The cells were then treated with MMPP for 24 h, and images were captured using an inverted phase-contrast microscope (magnification at 4). The wound areas were calculated and normalized with those at 0 h using Image J [19].

### Invasion Assay

The upper chamber was coated with 0.1% gelatin and 7% basement membrane extract (R&D Systems, USA). Cells were seeded in the upper chamber with serum-free medium, whereas the bottom chamber was filled with medium containing 10% FBS [20]. MMPP added to the upper chamber. The invading cells were stained with Diff-Quick (Sysmex, Japan). Images were obtained using a microscope (magnification at 4).

### Statistical Analysis

Statistical analysis was conducted using one-way ANOVA with Tukey’s honest significant difference test. Differences were considered significant at *p* < 0.05. Results were obtained from three independent experiments and are expressed as the mean ± standard deviation (SD).

## Results

### Effects on Cell Viability in BC Cell Lines

**Fig. 1:**
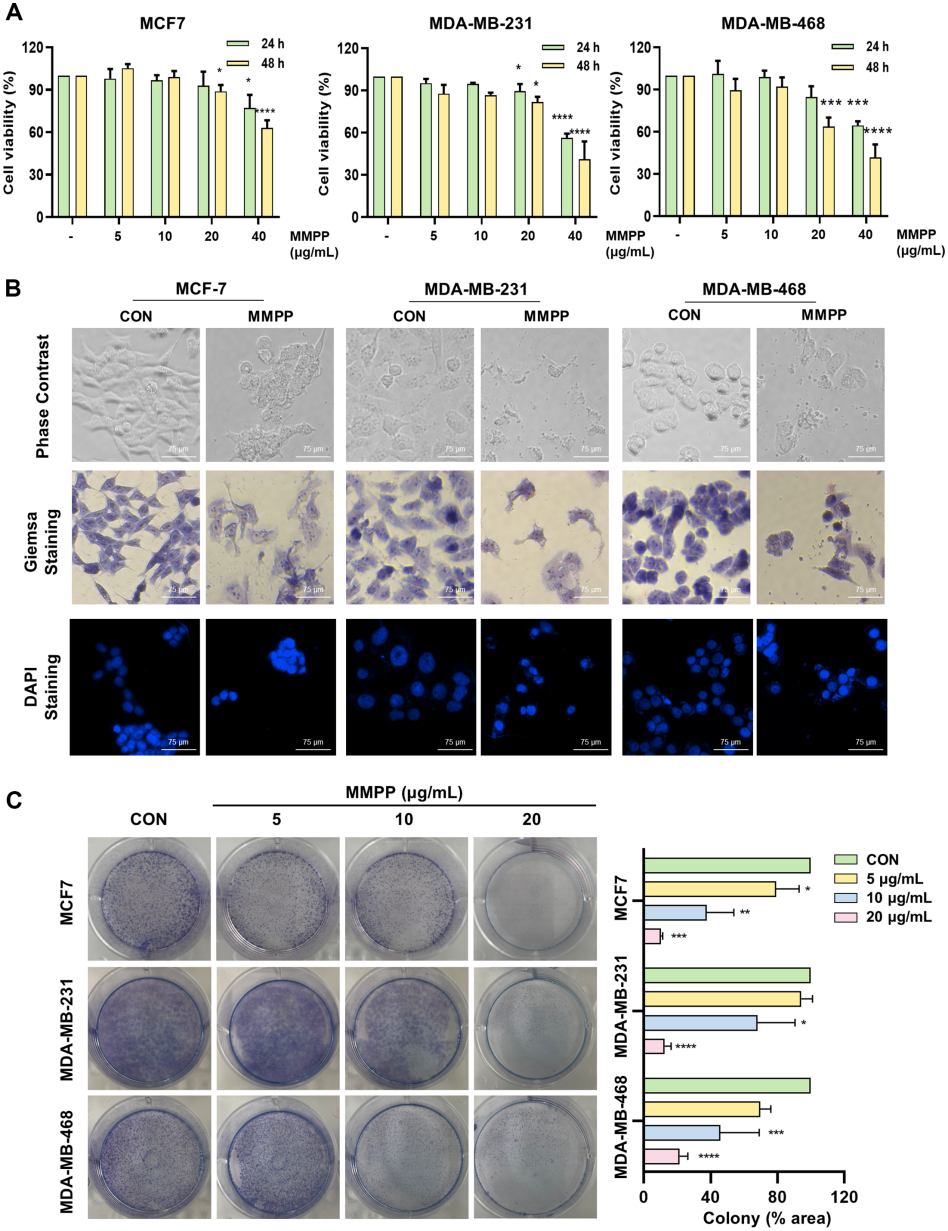
Effects on viability, morphology, nuclear condensation, and colony formation in BC cell lines. (**A**) MMPP (0, 5, 10, 20, and 40 μg/ml) treated in MCF7, MDA-MB-231, and MDA-MB-468 cells for 24 h or 48 h. The cell viability was evaluated by MTS assay, and then the cell viability (%) was normalized to control group. (**B**) The cells were treated with MMPP (40 μg/ml) for 24 h, subsequently stained with Giemsa and DAPI to observe morphological change and nuclear condensation. (magnification at × 20). Scale bar = 75 μm. (**C**) MMPP (0, 5, 10, and 20 μg/ml) treated for 2 days, and then transferred into fresh medium for 5 days. Colonies were stained with Giemsa and their area was measured and normalized to control group (*n* = 3). *p < 0.05, **p < 0.01, ***p < 0.001, ****p < 0.0001. *CON versus MMPP.

**Fig. 2:**
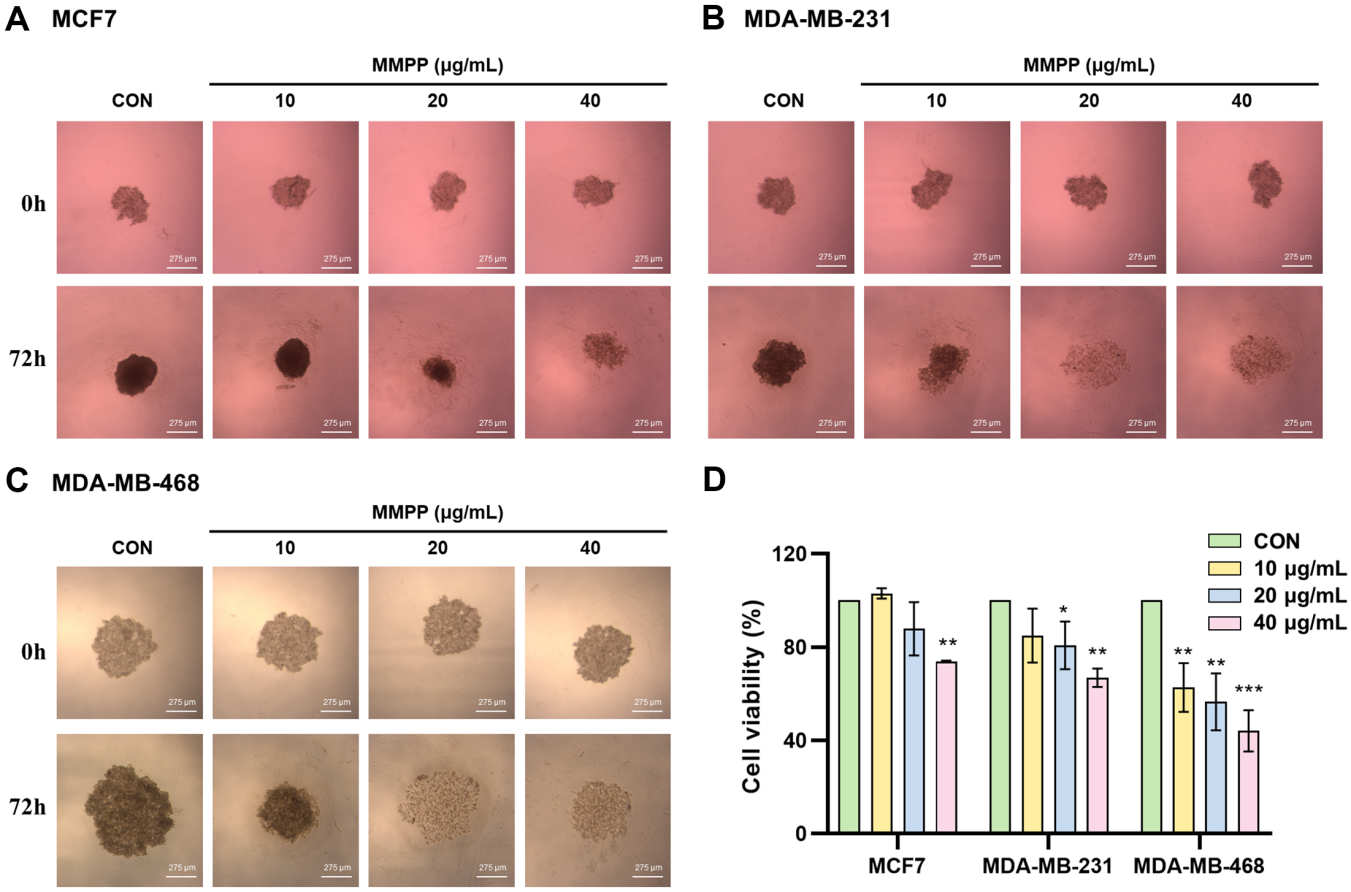
Effects on cell viability in 3D breast cancer cell cultures. Multicellular aggregates were formed after seeding in round-bottom 96-well plates for 48 h. MMPP (10, 20, and 40 μg/ml) treated in (**A**) MCF7, (**B**) MDA-MB-231, and (**C**) MDAMB- 468 for 72 h. (magnification at × 10). Scale bar = 275 μm. (**D**) Cytotoxicity of BC multicellular aggregates was assessed using MTS assay. The cell viability (%) was normalized to control group (*n* = 3). *p < 0.05, **p < 0.01, ***p < 0.001. *CON versus MMPP.

We treated with MMPP in BC cell lines and performed MTS assay to evaluate the effects of MMPP on cell viability. When exposed to an MMPP concentration of 40 μg/ml for 48 h, the cell viability in the MCF7, MDA-MB-231, and MDA-MB-468 cell lines decreased by 64.18%, 41.28%, and 41.87%, respectively. MMPP significantly declined cell viability in a dose-dependent manner (Fig. 1A). The half maximal inhibitory concentrations (IC50) in the MCF7, MDA-MB-231, and MDA-MB-468 cell lines were 63.13 μg/ml, 59.43 μg/ml, and 58.46 μg/ml, respectively. In contrast, the normal cell line HaCaT exhibited a lower reduction in cell viability, with only a 90.36%decrease at MMPP concentration of 40 μg/ml for 48 h (Fig. S1). Furthermore, MMPP induced morphological changes and nuclear condensation, which are characteristic hallmark of apoptosis (Fig. 1B). We also evaluated the proliferative activity of MMPP by observing colony formation. Our results indicated that MMPP suppressed colony formation in all BC cell liens (Fig. 1C). Breast cancer grows in not two-dimensional (2D) monolayer, but three-dimensional (3D) multilayer structure [4]. To attempt to compensate for this physiological aspect, we evaluate the cell viability in 3D multicellular aggregates of BC cells. According to the MTS assay, MMPP exhibited cytotoxicity in a dose-dependent manner in BC multicellular aggregates (Fig. 2). Thus, MMPP is potential as a selective agent for cancer cell inhibition.

### Effects on Apoptosis in BC Cell Lines

**Fig. 3:**
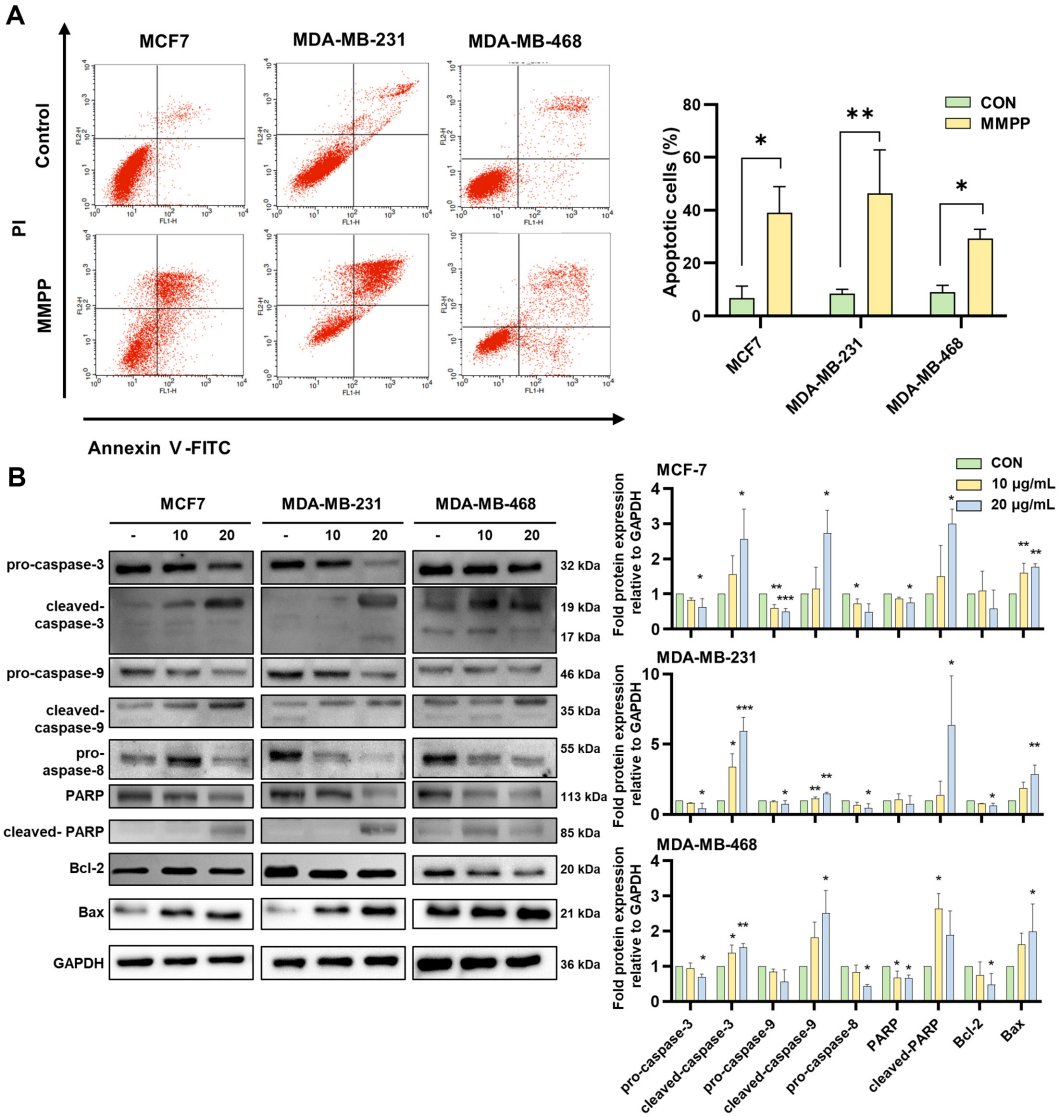
Effects on apoptosis in BC cell lines. (**A**) After treating with MMPP (40 μg/ml) for 24 h, harvested cells were labeled with AnnexinV/PI staining kit. (**B**) The cells were treated with MMPP (20 and 40 μg/ml) for 24 h, and then assessed by immunoblotting using antibodies for caspase-3, caspase-9, caspase-8, PARP, Bcl-2, Bax, and GAPDH. Bar graphs are represented band intensity and normalized with GAPDH (*n* = 3). *p < 0.05, **p < 0.01, ***p < 0.001. *CON versus MMPP.

To determine whether the MMPP-induced cytotoxicity was caused by apoptosis, we assayed Annexin V-PI staining. MMPP significantly promoted apoptosis of MCF7, MDA-MB-231, and MDA-MB-468 cells (Fig. 3A). Moreover, MMPP activated pro-apoptotic factors, such as caspase-3, caspase-8, caspase-9, and Bax, and downregulated the anti-apoptotic factor Bcl-2. In addition, MMPP induced PARP cleavage, which is a hallmark of apoptosis. (Fig. 3B).

### Effects on Migration and Invasion in BC Cell Lines

**Fig. 4:**
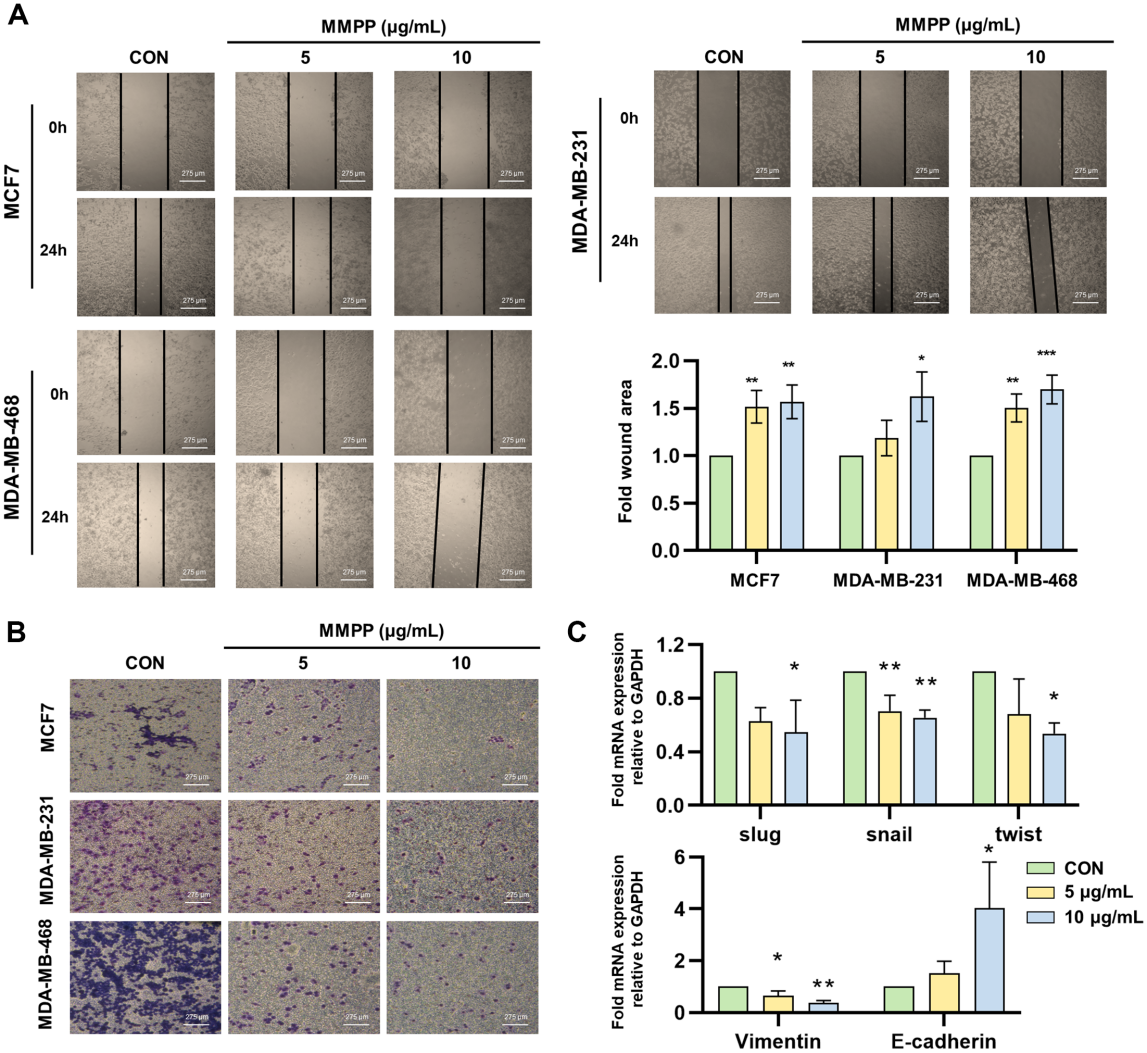
Effects on migration, invasion, and EMT transcription factor expression in BC cell lines. (**A**) MMPP (0, 5, and 10 μg/ml) treated in BC cell lines for 24 h in serum-free medium. The migration abilities were analyzed by wound healing assay. (magnification at × 10). Scale bar = 275 μm. (**B**) MMPP (0, 5, and 10 μg/ml) treated in BC cell lines for 24 h. Invasion assay was performed, and then invasive cells were stained with Diff-Quick staining kit. (magnification at × 10). Scale bar = 275 μm. (**C**) MMPP (0, 5, and 10 μg/ml) treated in BC cell lines for 24 h. mRNA expressions of slug, snail, twist, vimentin, and Ecadherin were examined using RT-PCR in MDA-MB-231. The mRNA levels were normalized with GADPH compared by control group (*n* = 3). *p < 0.05, **p < 0.01, ***p < 0.001. *CON versus MMPP.

We confirmed the migratory and invasive abilities of MMPP at non-cytotoxic concentrations of 5 and 10 μg/ml. In wound healing and invasion assays, MMPP suppressed the migration and invasion in BC cell lines (Fig. 4A and 4B). The mRNA levels of slug, snail, twist, and vimentin were significantly downregulated by MMPP treatment in MDA-MB-231 cells, while E-cadherin was upregulated by MMPP (Fig. 4C).

### Effects on VEGFR2/AKT and PPARγ/PTEN/AKT Pathway in BC Cell Lines

**Fig. 5:**
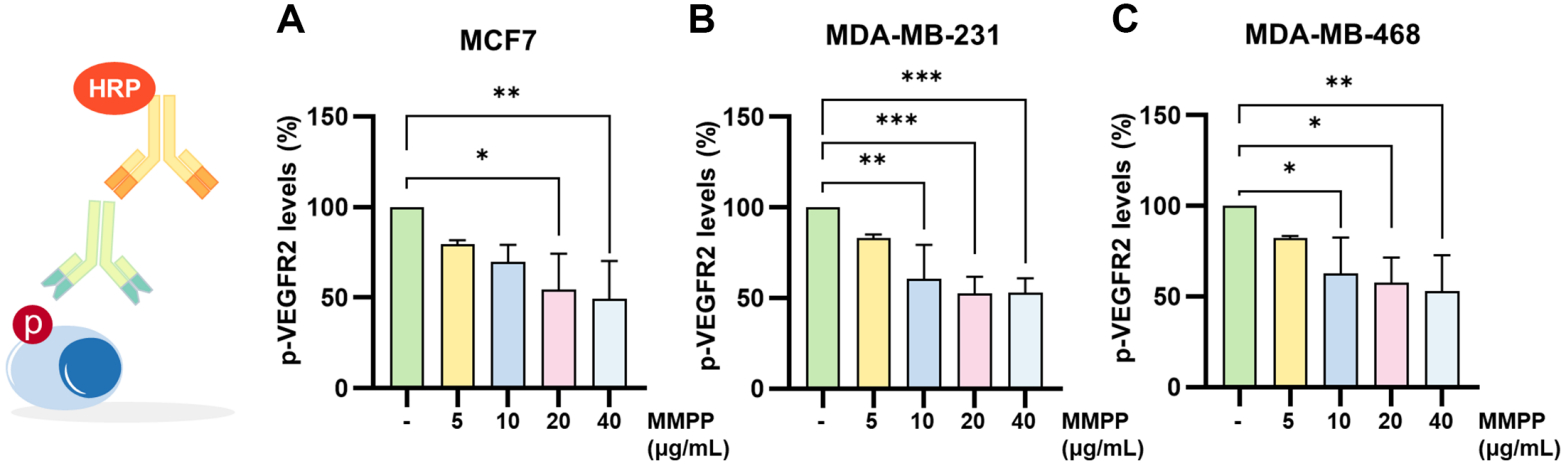
Effects on p-VEGFR2 levels in BC cell lines. After seeding in 96-well plates, MMPP (0, 5, and 10 μg/ml) treated in (**A**) MCF7, (**B**) MDA-MB-231, and (**C**) MDA-MB-468 for 30 min. Cell-based ELISA was performed using p-VEGFR2 (Tyr 1175) antibody to measure phosphorylation of VEGFR2. The absorbance was analyzed at 450 nm, and normalized to control group (*n* = 3). *p < 0.05, **p < 0.01, ***p < 0.001. *CON versus MMPP.

**Fig. 6:**
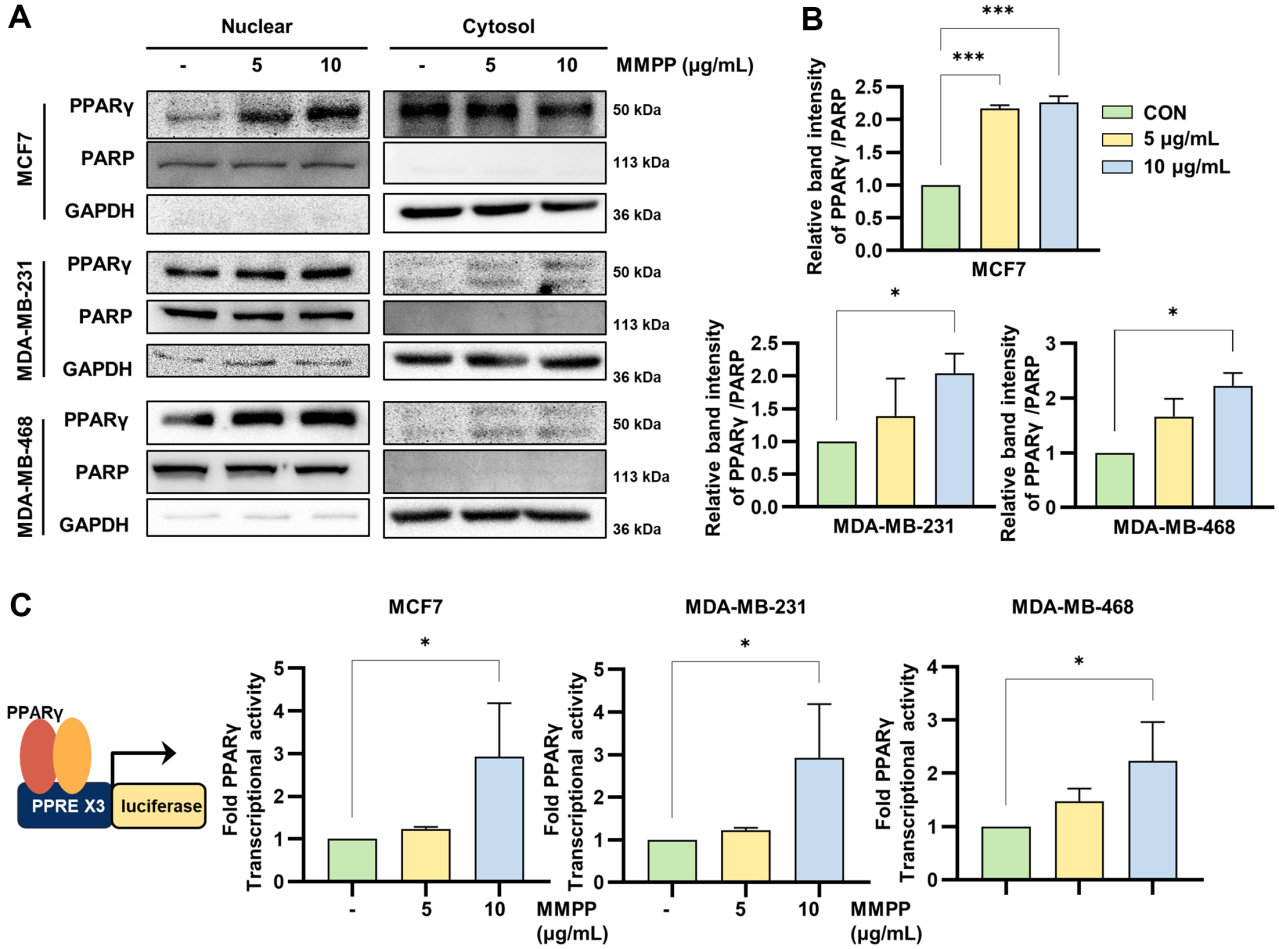
Effects on PPARγ translocation and transcriptional activity in BC cell lines. (**A**) PPARγ translocations were analyzed by immunoblotting of the nucleic and cytosolic extracts. MMPP (0, 5, and 10 μg/ml) treated in BC cell lines for 2 h. (**B**) Bar graphs are represented band intensity and normalized with PARP in nuclear fraction. (**C**) PPARγ transcriptional activities were determined by luciferase assay. The cells were transfected with (PPRE3)-tk-luciferase construct and pRL control vector. Various concentrations of MMPP were treated into transfected cells for 24 h, and then conducted luciferase assay. Fold PPARγ transcriptional activities were normalized to control group (*n* = 3). *p < 0.05, ***p < 0.001. *CON versus MMPP.

**Fig. 7:**
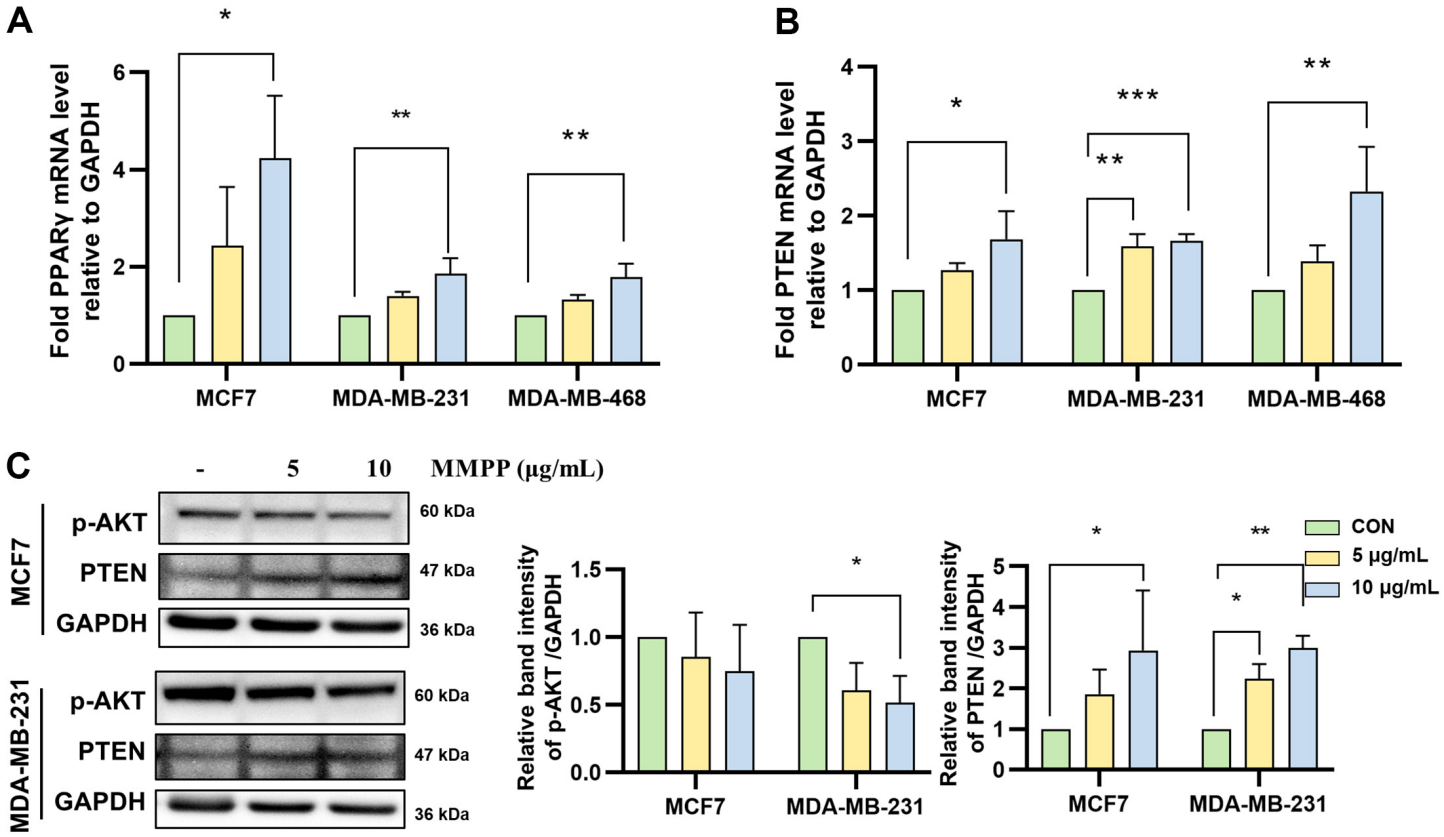
Effects on PPARγ/PTEN/AKT pathway in BC cell lines. mRNA levels of (**A**) PPARγ (**B**) PTEN were assessed by RT-PCR. MMPP (0, 5, and 10 μg/ml) treated in BC cell lines for 24 h. The mRNA levels were normalized with GADPH compared by control group. (**C**) Protein expressions of p-AKT and PTEN were measured by immunoblotting in MCF7 and MDA-MB-231 cells. Bar graphs are normalized with GAPDH (*n* = 3). *p < 0.05, **p < 0.01, ***p < 0.001. *CON versus MMPP.

We investigated that MMPP could regulate VEGFR2 and PPARγ in BC cell lines. We treated MMPP for 30 min in BC cell lines, followed by conducting cell-based ELISA to measure the levels of anti-pVEGFR2 (Tyr 1175). The results demonstrated that MMPP significantly suppressed the phosphorylation of VEGFR2 by 49.50%, 52.84%, and 52.79% in each of the cell lines at their respective final concentration of MMPP (Fig. 5). To analyze whether MMPP regulates PPARγ, we examined translocation and transcriptional activity of PPARγ. MMPP significantly increased PPARγ translocation to the nucleus (Fig. 6A and 6B). MMPP upregulated the transcriptional activity of PPARγ (Fig. 6C). Additionally, mRNA expression of PPARγ and PTEN was increased by MMPP in BC cell lines (Fig. 7A and 7B). MMPP treatment increased the protein expressions of PTEN and decreased those of p-AKT (Fig. 7C). Our findings suggested that MMPP regulates the VEGFR2/AKT and PPARγ/PTEN/AKT pathways.

## Discussion

Compared to traditional agents, investigation for multiple targeted drugs in clinical oncology has been driven by their superior efficacy, due to the multiple signaling pathways controlled the cancer progression [21]. These drugs target multiple pathways responsible for cancer progression, including cancer cell proliferation, apoptotic regulation, angiogenesis, and metastasis [22]. However, the effect of dual-targeting drugs for VEGFR2 and PPARγ in breast cancer remains unclear. This study demonstrates that MMPP is a dual-targeting drug for VEGFR2 and PPARγ, which stimulates apoptosis and restrains survival, migration, and invasion in breast cancer.

Breast cancer is classified into different subtypes based on the expression of receptors such as estrogen receptor (ER), progesterone receptor (PR), and human epidermal growth factor receptor 2 (HER2) [23]. The main subtypes include luminal (ER^+^/PR^+^/HER2^-^), HER2 (ER^-^/PR^-^/HER2^+^), and TNBC (ER^-^/PR^-^/HER2^-^) [24]. Targeted therapies were invented for luminal and HER2 types due to the presence of specific receptors. However, TNBC types are challenging to develop targeted therapies and are commonly managed by systemic chemotherapy [25]. Therefore, the investigation of effective treatment for TNBC remains as important area of research. In previous study, we demonstrated that MMPP possesses the ability to selectively target VEGFR2 and PPARγ [11, 12]. Interestingly, both VEGFR2 and PPARγ have been identified as targets for BC treatment. Based on this knowledge, we hypothesized that MMPP may exhibit anticancer effects by dual-targeting VEGFR2 and PPARγ in BC cell lines, including both luminal and TNBC subtypes.

VEGFR2 not only acts as a key regulator for angiogenesis in endothelium but is also highly expressed in BC, where it participates in cancer survival and metastasis [26, 27]. Hence, VEGFR2 has emerged as a novel target for treating TNBC [28]. Autophosphorylation of VEGFR2 activates PI3K/AKT pathway, which in turn trigger downstream pathway involved in proliferation, migration, and invasion [29]. Our results revealed that MMPP suppressed VEGFR2 kinase activity in breast cancer cell lines.

PPARγ, a ligand-activated nuclear receptor, has an important role in regulating adipogenesis [30]. Once activated, PPARγ translocates into the nucleus and binds to peroxisome proliferator response elements (PPREs), inducing the transcription of targets including PTEN [31]. PPARγ ligands trigger apoptosis by upregulating PTEN in BC cells [7]. PTEN is important tumor suppressors that dephosphorylates phosphatidylinositol-3,4,5-triphosphate (PIP3), and then these signaling inactivates AKT [32]. Our results indicated that MMPP binds to PPARγ, involving in the activation and translocation of PPARγ. Promotion of PPARγ transcriptional activity upregulates mRNA and protein expressions of PTEN in BC cells. The inhibition of VEGFR2 and upregulation of PTEN induced by MMPP suppressed AKT phosphorylation. AKT is a serine-threonine kinase that induces tumor-associated processes, including proliferation, survival, EMT, and angiogenesis. AKT pathway is frequently overexpressed in cancer, resulting in failure to regulate apoptosis, which leads to uncontrolled cell growth [33, 34]. AKT pathway regulates apoptosis through the caspase and Bcl-2 families [35]. The Bcl-2 family consists of anti-apoptotic (Bcl-2) and pro-apoptotic proteins (Bax) [36]. Caspases have essential roles in the regulation of apoptosis. Caspase-9 is an initiator of apoptosis that is inhibited by AKT, promoting the cleavage of caspases-3 [37]. Effector caspases activate various substrates, disrupt DNA and cellular components, and ultimately induce morphological shifts including cell shrinkage, nuclear condensation, and cell fragmentation [38]. MMPP treatment induced cell shrinkage and nuclear condensation. MMPP promoted apoptosis in breast cancer cells via cleavage of caspase-3, caspase-8, and caspase-9 and regulation of Bcl-2 and Bax through the VEGFR2/AKT and PPARγ/PTEN/AKT pathways. Additionally, the AKT pathway has a fundamental role in promoting metastasis by regulating EMT-promoting transcription factors, such as snail, slug, and twist [39, 40]. Our findings indicated that MMPP suppresses migration and invasion by downregulating EMT-promoting transcription factors in breast cancer cells.

Overall, MMPP has high cytotoxicity and significant potential in the treatment of BC. MMPP induces cancer cells apoptosis and inhibits migration/invasion via VEGFR2/AKT and PPARγ/PTEN/AKT pathways. In cases of TNBC, which poses challenges in targeted therapy, the high expression of both PPARγ and VEGFR2 provides opportunity for MMPP to be a promising treatment option. Therefore, MMPP is ascertained to be a potent anticancer drug for breast cancer by regulating VEGFR2 and PPARγ.

## Abbreviation

VEGFR2, vascular endothelial growth factor receptor 2; PPARγ, peroxisome proliferator-activated receptor gamma; PTEN, phosphatase and tensin homolog deleted on chromosome 10; MMPP, (E)-2-methoxy-4-(3-(4-methoxyphenyl)prop-1-en-1-yl) phenol; EMT, epithelial-mesenchymal transition; BC, breast cancer

## Supplemental Materials

Supplementary data for this paper are available on-line only at http://jmb.or.kr.
