# Supplementary material for: (E)-2-Methoxy-4-(3-(4-Methoxyphenyl) Prop-1-en-1-yl) Phenol Suppresses Breast Cancer Progression by Dual-Regulating VEGFR2 and PPARγ
Source: J Microbiol Biotechnol. 2023 Nov 3;34(2):240–8. doi: 10.4014/jmb.2309.09019 (PMC10940741; doi:10.4014/jmb.2309.09019)
Supplement: Supplementary file 1 [file jmb-34-2-240-supple.pdf]

J. Microbiol. Biotechnol.
https://doi.org/10.4014/jmb.2309.09019

Supplementary Table and Figure

(E)-2-methoxy-4-(3-(4-methoxyphenyl) prop-1-en-1-yl) phenol rt ooqdrrdr aqd` rs b` mbdq progression by dual-targeting VEGFR2 and PPARγ

Na-Yeon Kim[1], Hyo-Min Park[1], Hee Pom Lee[2], Jin Tae Hong[2], Do-Young Yoon[1*]

[1] Department of Bioscience and Biotechnology, Konkuk University, Seoul, Republic of Korea

[2] College of Pharmacy & Medical Research Center, Chungbuk National University, Cheongju, Republic of Korea

[*] Correspondence author

E-mail address: ydy4218@konkuk.ac.kr

MATERIALS AND METHODS

Cell culture

HaCaT cell lines were cultured in DMEM supplemented with 10 % (v/v) thermally inactivated fetal bovine serum (Hyclone Laboratories, Logan, UT, USA). The cells were incubated at 37°C and 5 % CO_2.

Cell viability assay

Cell viability was assessed using the 3-(4,5-dimethylthiazol-2-yl)-5-(3-carboxymethoxy phenyl)-2-(4-sulfophenyl)-2H-tetrazolium (MTS) and CellTiter 96 Aqueous One Solution Assays (Promega, Madison, WI, USA). Briefly, 20 µL MTS reagent was added to each well and incubated for 1 h. The absorbance was measured at 492 nm using a microplate reader (Apollo LB9110; Berthold Technologies GmbH, Bad Wildbad, Germany).

Supplementary Table

Table S1. Primer sequences.

Genes	Primer	Sequences (5'-3')
PPARγ	Forward	5′-GTACTGTCGGTTTCAGAAGTGCC-3′
	Reverse	5′-ATCTCCGCCAACAGCTTCTCCT-3′
PTEN	Forward	5′-TGAGTTCCCTCAGCCGTTACCT-3′
	Reverse	5′-GAGGTTTCCTCTGGTCCTGGTA-3′
Snail	Forward	5'-TGCCCTCAAGATGCACATCCGA-3'
	Reverse	5'-GGGACAGGAGAAGGGCTTCTC-3'
Slug	Forward	5'-ATCTGCGGCAAGGCGTTTTCCA-3'
	Reverse	5'-GAGCCCTCAGATTTGACCTGTC-3'
Twist	Forward	5'-GCCAGGTACATCGACTTCCTCT-3'
	Reverse	5'-TCCATCCTCCAGACCGAGAAGG-3'
E-cadherin	Forward	5’-TGCCATTCTGGGGATTCT-3’
	Reverse	5’-TGGCTCAAGTCAAAGTCCTG-3’
Vimentin	Forward	5’-GGATGTTACAATGCGTCTC-3’
	Reverse	5’-TGCTGTTCCTGAATCTGAGC-3’
GAPDH	Forward	5′-ATGGGGAAGGTGAAGGTCGG-3′
	Reverse	5′-CCTTGGAGGCCATGTGGGCC-3′

Supplementary Figure

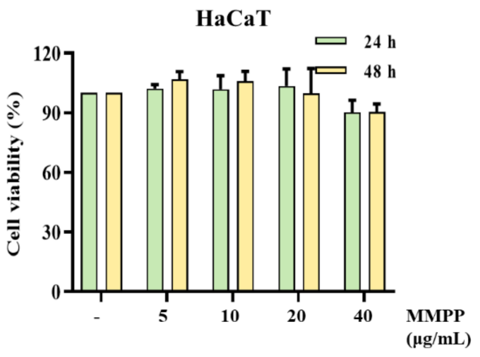

Fig. S1. Effects of MMPP on cell viability in HaCaT cell lines. HaCaT cell lines were treated with MMPP (5, 10, 20, and 40 μg/mL) for 24 hours or 48 hours. Cell viability was assessed by MTS assay (n = 3).
